# Acne Vulgaris

**Published:** 1905-04-15

**Authors:** 


### ACNE VULGARIS.

Acne shares with an unfortunately large collection of skin diseases a frequent disregard for treatment. This quality is particularly unlucky in a disease which so commonly selects the face as the chief sphere of its activity, and which, in addition to the disfigurement attending its active phase, not infrequently leaves a permanent legacy of scars to mark its sojourn. Dr. G. T. Jackson[1] seeks to combat the pessimistic attitude of the profession, which, regarding acne as a self-limited disease of early adult life, is content to exercise a kind of vicarious resignation and wait for the passage of years to achieve the natural elimination of the malady. He avers that, once the fundamental nature of the affection is properly appreciated, a reasonable perseverance in treatment will cure a majority of cases.

The elementary factors in acne are a superactivity of the sebaceous glands (evidenced by the greasy skin always associated with the lesion), a plugging of the orifices of the sebaceous ducts, and, finally, an infection of the collected sebum by micro-organisms (especially staphylococci), leading to pustulation. Some authorities, notably Sabouraud and Gilchrist, hold that the plugging of the ducts depends upon a specific bacillus. Whether this be the fact or not, there is little doubt that the accumulation of the sebum and its subsequent conversion into pus are two entirely distinct lesions, the latter depending upon an accident of infection, and playing no part in the normal evolution of sebaceous collections. This seems clear from the fact that an accumulation of sebum in the sebaceous glands themselves, as in the case of milium, may persist indefinitely without suppuration.

The treatment recommended by Dr. Jackson is both general and local; the general treatment merely consists of means to increase the bodily health, such as a careful dietary and avoidance of stimulants and unhygienic habits, though we fancy the author would be put to it to demonstrate convincingly a connection between tight-lacing and acne. The local treatment is directed in the first place against the accumulation of sebum, and in the second against the possibility of microbic infection. The first indication is met by the author in a somewhat heroic fashion by going over the affected surface with a curette and scraping out all the spots, subsequently applying pure carbolic acid to prevent reaccumulation. The second indication is met by the use of antiseptic lotions and frequent cleansing with soap.

Malcolm Morris [2] advises that when suppuration has not yet occurred the comedones should be squeezed out and the parts affected washed frequently and energetically with soft soap and a coarse flannel. The skin is then to be kept disinfected by the application of sulphur ointment (gr. 10 to the ounce) or resorcin (gr. 15 to the ounce) in equal parts of lanoline and vaseline. Suppuration must be treated by incision and evacuation, as in any other part of the body.

One other treatment of acne has lately achieved prominence in the hands of Professor A. E. Wright.[3] It consists in developing in the patient an artificial immunity to the staphylococci associated with the pustular stage of acne by the injection of staphylococcus vaccines. This treatment, we understand, does not affect the accumulations of sebum, but prevents their conversion into pustules. Malcolm Morris [4] in a recent address expresses the opinion that the use of such "heavy artillery" is not called for by the trifling nature of the malady, especially as the suppuration-element of the disease can be met by antiseptics. The conclusion of the matter would appear to be this. That perseverance in the local treatment of acne is successful when the patience of the victim is coextensive with the long duration and somewhat painful character of the necessary remedial measures. This patience is probably directly proportional to the victim's æsthetic sense, or personal vanity. A man who is regardless of his personal appearance is not likely to submit to a trying ordeal in order to rid himself of a malady which affects his comfort to a very inconsiderable degree, while to the opposite type the disfigurement may make the disease a misfortune grave enough to prompt the requisite sacrifices. The gravity and curability of the affection can therefore be estimated only in relative terms.

[1] Med. Rec., March 18, 1905. [2] Diseases of the Skin. [3] Clin. Jour., Nov. 9, 1904. [4] Brit. Med. Jour., April 1, 1905.

